# Supplementary material for: Association between oral microbiome and seven types of cancers in East Asian population: a two-sample Mendelian randomization analysis
Source: Front Mol Biosci. 2023 Nov 21;10:1327893. doi: 10.3389/fmolb.2023.1327893 (PMC10702768; doi:10.3389/fmolb.2023.1327893)
Supplement: Supplementary file 1 [file DataSheet1.ZIP › Supplementary Materials/MR plots of saliva/saliva═╝/Prostate cancer/pheno.722_to_prostate cancer_forest.pdf]

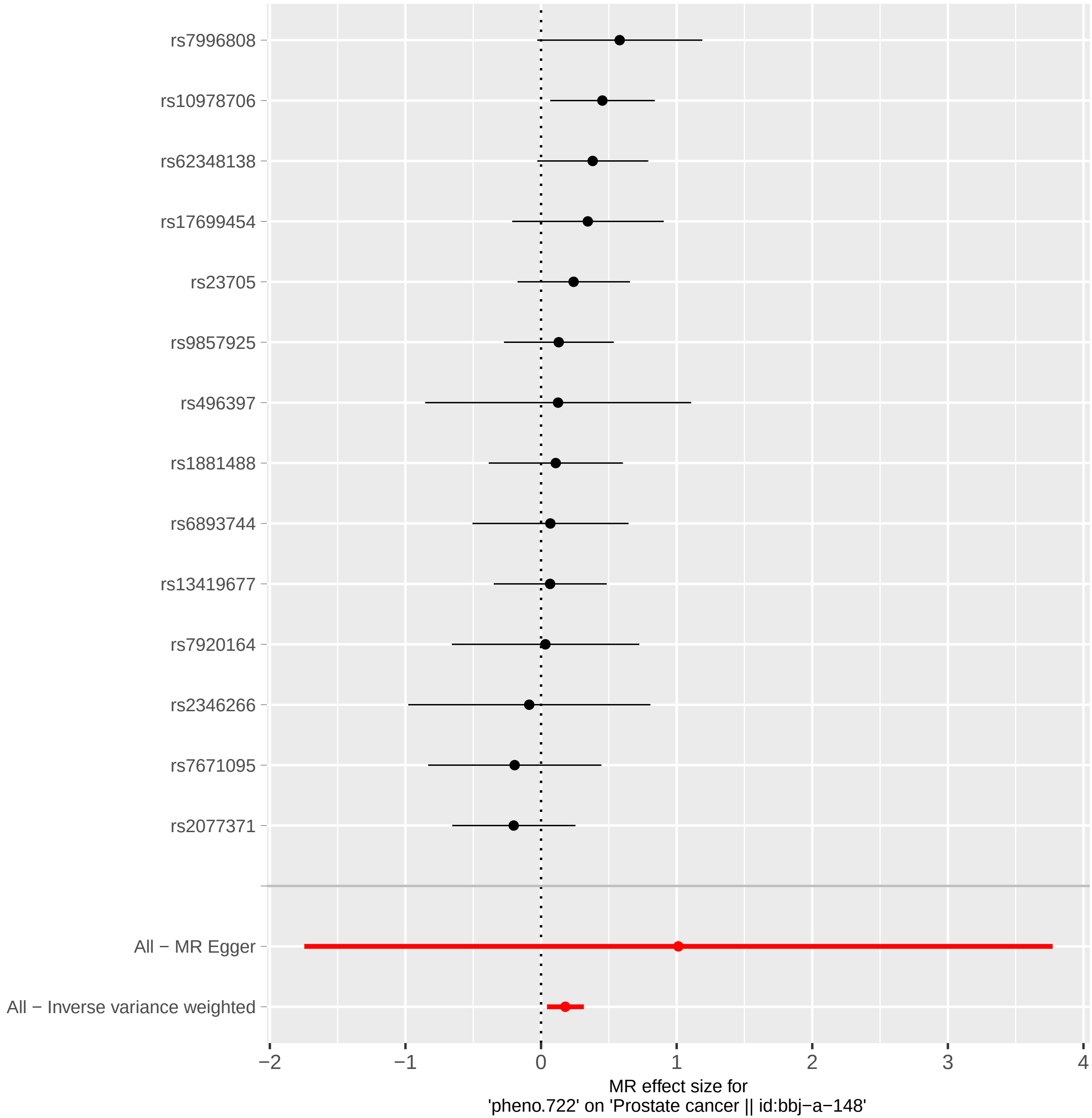
rs7996808
rs10978706
rs62348138
rs17699454
rs23705
rs9857925
rs496397
rs1881488
rs6893744
rs13419677
rs7920164
rs2346266
rs7671095
rs2077371
All − MR Egger
All − Inverse variance weighted
−2
−1
0
1
2
3
4
MR effect size for
'pheno.722' on 'Prostate cancer || id:bbj−a−148'
